# Emerging Object Representations in the Visual System Predict Reaction Times for Categorization

**DOI:** 10.1371/journal.pcbi.1004316

**Published:** 2015-06-24

**Authors:** J. Brendan Ritchie, David A. Tovar, Thomas A. Carlson

**Affiliations:** 1 Department of Philosophy, University of Maryland, College Park, Maryland, United States of America; 2 Perception in Action Research Centre, Department of Cognitive Science, Macquarie University, Sydney, New South Wales, Australia; 3 ARC Centre of Excellence in Cognition and its Disorders, Macquarie University, Sydney, New South Wales, Australia; 4 School of Medicine, Vanderbilt University, Nashville, Tennessee, United States of America; University of Tübingen and Max Planck Institute for Biologial Cybernetics, GERMANY

## Abstract

Recognizing an object takes just a fraction of a second, less than the blink of an eye. Applying multivariate pattern analysis, or “brain decoding”, methods to magnetoencephalography (MEG) data has allowed researchers to characterize, in high temporal resolution, the emerging representation of object categories that underlie our capacity for rapid recognition. Shortly after stimulus onset, object exemplars cluster by category in a high-dimensional activation space in the brain. In this emerging activation space, the decodability of exemplar category varies over time, reflecting the brain’s transformation of visual inputs into coherent category representations. How do these emerging representations relate to categorization behavior? Recently it has been proposed that the distance of an exemplar representation from a categorical boundary in an activation space is critical for perceptual decision-making, and that reaction times should therefore correlate with distance from the boundary. The predictions of this distance hypothesis have been born out in human inferior temporal cortex (IT), an area of the brain crucial for the representation of object categories. When viewed in the context of a time varying neural signal, the optimal time to “read out” category information is when category representations in the brain are most decodable. Here, we show that the distance from a decision boundary through activation space, as measured using MEG decoding methods, correlates with reaction times for visual categorization during the period of peak decodability. Our results suggest that the brain begins to read out information about exemplar category at the optimal time for use in choice behaviour, and support the hypothesis that the structure of the representation for objects in the visual system is partially constitutive of the decision process in recognition.

## Author Summary

Recognizing an object in the world (e.g. a cat) takes just a fraction of a second. Recent advances in neuroscience have allowed researchers to measure the emergence and dynamics of time-varying neural signals that allow us to quickly recognize objects visually. At each moment in time, these neural signals can be characterized as patterns of neural activity that cluster categorically in a high-dimensional activation space. Within this space, a boundary can be drawn between the clusters of activity patterns, which can then be used to discriminate object categories (e.g. cats vs. cars). Based on which side of the boundary a pattern falls, researchers can guess (or “decode”) the category membership of an object that an observer is viewing. In the present study, we provide evidence that at the time when category clusters are best separated in activation space—that is, the time when category information is best suited to be “read out” from the brain’s signals—the structure of activation space can be used to predict behaviour. Our results provide insights into both when and how the brain’s representational architecture supports rapid object recognition.

## Introduction

When recognizing objects the brain does not take its time. Although object recognition is one of the most computationally difficult feats performed by the visual system, it is carried out in an ultra-rapid fashion, with relative ease and high fidelity [1–2]. Some of the most convincing evidence for ultra-rapid recognition comes from behavioural research. Using saccadic eye-movements, subjects can reliably categorize object exemplars as quickly as 120 ms post-stimulus onset [3–4] and faces as fast as 100 ms [5]. The rapidity of saccadic reaction times for categorization suggests that information about stimulus category must be available in the brain very shortly after stimulus onset [3, 6].

The application of multi-variate pattern analysis (MVPA), or “decoding”, methods to time-series data has allowed researchers to characterize the emergence and dynamics of time-varying neuronal activity associated with objects in the brain. Concordant with the early availability of category information observed in behavioural research, information about object category can be decoded as early as 60–100 ms after stimulus onset [7–10]. While decoding onset is largely stable across categories, MEG decoding studies have found that peak decoding varies with the hierarchical organization of object categories; classifiers trained to discriminate subordinate categories (e.g. face, body) peak earlier in their performance relative to ones trained to discriminate superordinate categories (e.g. animacy) [7–8]. These results suggest that peak decoding indexes the optimal time to read out information about stimulus categories [7], and that time-resolved decoding provides a method for revealing the representational dynamics of the brain [11].

The classifiers used in decoding analysis rely on boundaries through high-dimensional activation spaces that separate patterns of activity for object exemplars based on category (e.g. faces vs. houses). Classifier performance is better when an activation space is organized along clear categorical dimensions. For example, object category is highly decodable from the activity patterns in human and monkey inferior temporal cortex (IT), a region strongly implicated in representing object categories [12], but only moderately decodable in early visual cortex [13], which encodes low-level image features (e.g. edges and colors). With time-resolved decoding, classifier performance improves at time points when activation spaces are better organized along categorical dimensions. If these spaces indeed reflect underlying representational dynamics, then an important question is when and how these emergent activation spaces are used by the brain in a task related manner.

**Fig 1:**
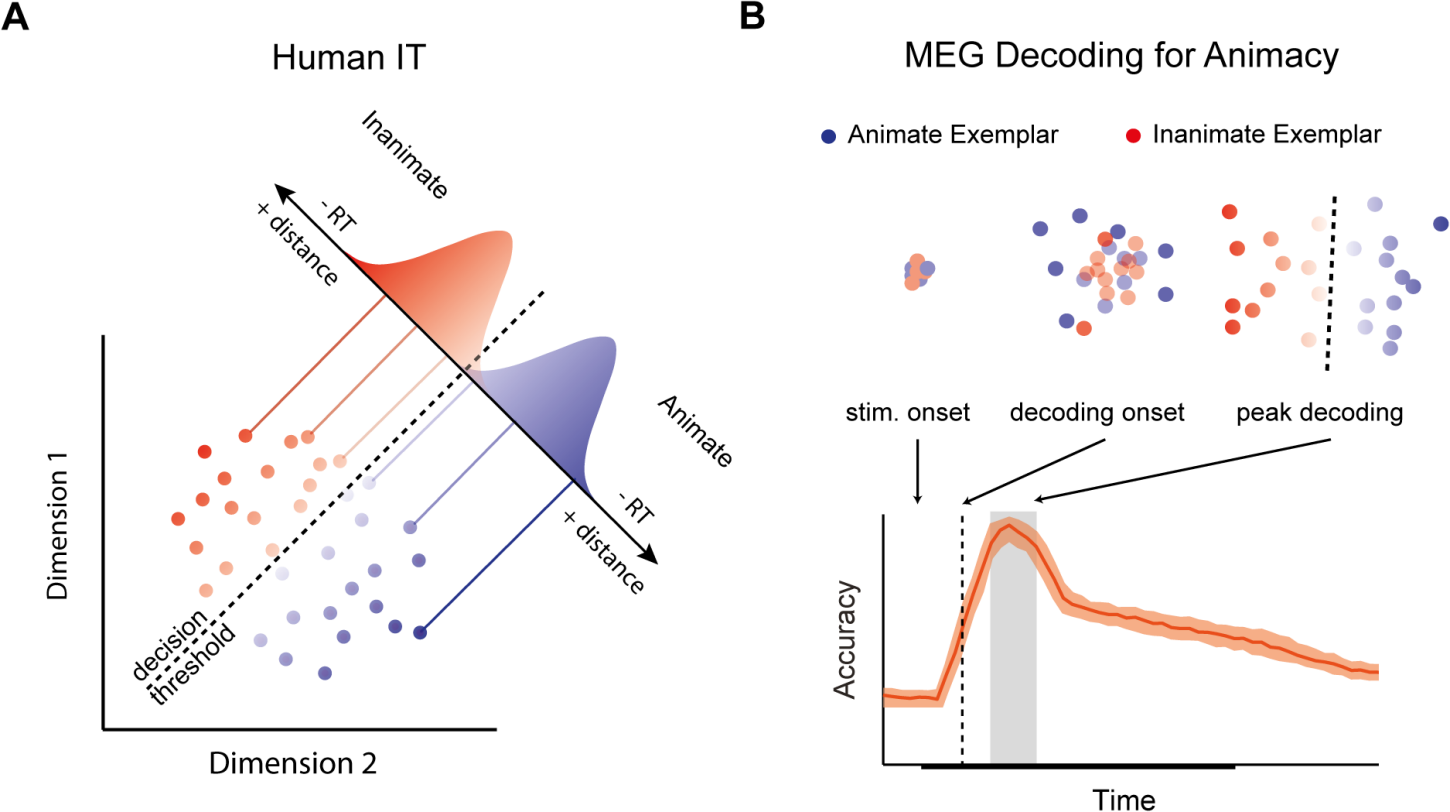
Distances from a decision boundary through activation space can be used to predict reaction times (RT). (A) A hypothetical 2D activation space for human IT representing animate and inanimate object exemplars. Activation patterns for individual exemplars are projected onto a discriminant axis, which differentiates patterns based on animacy. A decision boundary placed along the axis allows for classification of animate and inanimate exemplars. Gaussian distributions along the discriminant axis reflect “decision noise”. Exemplar representations closer to the boundary produce more ambiguous evidence compared to exemplar representations far from the boundary. An implication of classic signal detection theory is that RTs will correlate negatively with distance from the boundary. (B) A hypothetical emergent activation space for animate vs. inanimate object exemplars as would be revealed using MEG decoding methods. Stimulus onset is the time the stimulus is presented. The decoding onset (dashed line) is the first time point that a classifier trained to discriminate between animate and inanimate examplars performs significantly above chance. Peak decoding (gray box) is the optimal time point to read out information about stimulus category. Clusters depict the hypothetical 2D activation spaces at notable points in the decoding time-course.

One approach to forging a link between decodability and behaviour holds that a boundary that separates object exemplars based on category membership (e.g. animate vs. inanimate) in an activation space reflects a *decision* boundary for behavioural categorization (Fig 1A; [14]). Signal detection theory [15] suggests that evidence close to a decision boundary is more ambiguous, while evidence far from the boundary is less ambiguous. Since decision time tends to increase with the quality of evidence for an observer, and ambiguity is one dimension of evidence quality, a simple consequence of this familiar picture from classic psychophysics is that reaction times will correlate negatively with distance from a decision boundary: the farther an object representation is from the decision boundary through the space, the less ambiguous the evidence, and the faster the reaction times [16–18]. Using fMRI, Carlson et al. [14] tested this distance hypothesis using an activation-space for objects constructed from patterns of activity in human IT. They found that distance from a categorical boundary for animacy through the activation space of the region negatively correlated with RTs, suggesting that object representations form part of the decision-process for visual categorization (cf. [19]).

In the present study, we show *when* distances from a boundary through a high-dimensional activation space is predictive of reaction times, in order to reveal a link between the time-varying signals revealed by MEG decoding and behaviour. If peak decoding does indeed index the optimal time to read-out information about object category, then a plausible hypothesis is that peak decoding is the time at which the brain constructs a representation of the stimulus that is predictive of reaction time behaviour (Fig 1B). We tested this hypothesis by examining the relationship between emergent activation spaces for objects in the brain, measured using MEG, and reaction times for object categorization. Controlling for potential task-related and motor confounds, our study shows that reaction times begin to correlate with representational distances during peak decoding, and that the relationship between representational distance and reaction times in general follows the time-course of decoding. Our results provide support for the hypothesis that the brain reads out information when sensory evidence is optimal for making a decision about object category.

## Results

**Fig 2:**
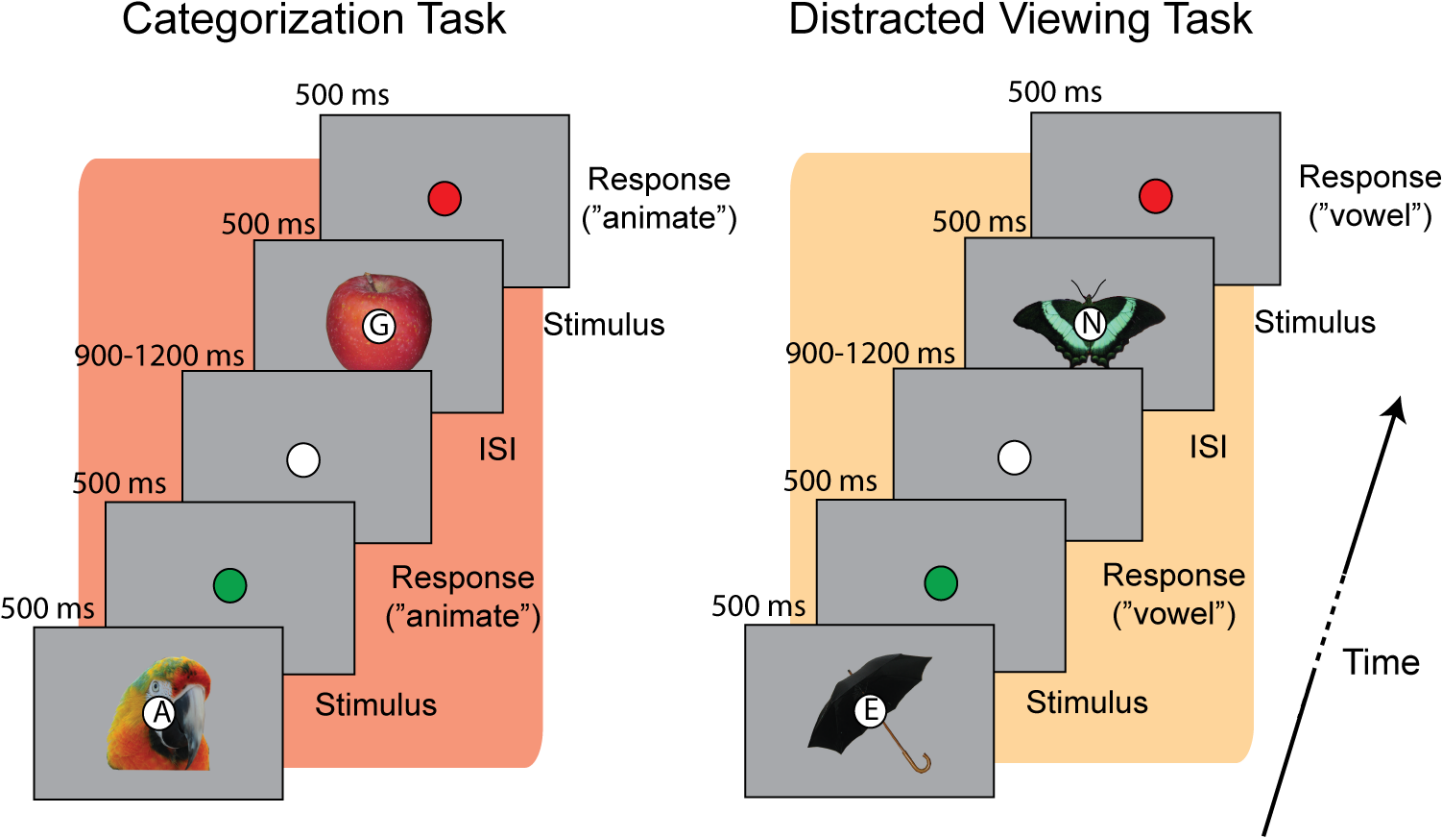
Behavioral paradigm. Trial structure for the two experimental tasks. On each trial a letter (vowel or consonant) was superimposed on the fixation circle in the centre of each object exemplar. When performing the *categorization* task subjects judged whether the exemplar stimulus was animate or inanimate, while during the *distracted viewing* task subjects judged whether the letter was a vowel or consonant. After subjects responded the fixation circle briefly flashed green (correct response) or red (incorrect response or no response) to provide trial-by-trial feedback on performance.

Subjects were shown a series of images while their brain activity was recorded using MEG. Each image depicted either an animate or inanimate object from a set of twenty-four object exemplars (Fig 2). Superimposed onto each image was a letter at fixation. We focused on the distinction between animate vs. inanimate exemplars because it is reliably decoded with MEG [7–8], and because it was the same distinction relied on in previous work that measured the correlation between representational distance and reaction time [14]. In separate blocks of trials subjects either actively categorized images as animate or inanimate (*categorization* task), or responded whether the letter at fixation was a vowel or consonant (*distracted viewing* task), while passively viewing the exemplar stimuli (Fig 2).

### Behavioral performance

Reaction time (RT) and choice behaviour were recorded on each trial during the scanning session. Overall subjects performed well at both tasks. Mean accuracy for the categorization task was 91.8% (*SD* = 5.6%) correct, and 87.2% (*SD* = 8.5%) correct for the distracted viewing task. The mean RTs were 469 ms (*SD* = 82 ms) for the categorization task, and 516 ms (*SD* = 96 ms) for the distracted viewing task.

### Decoding animacy from MEG time series

**Fig 3:**
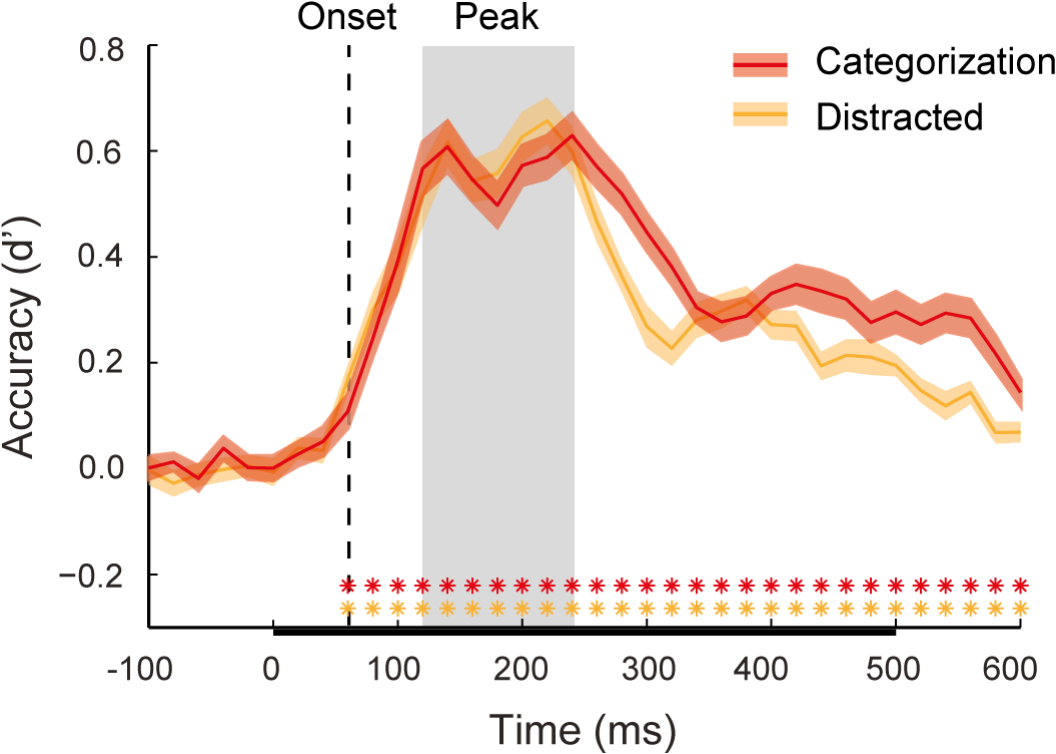
MEG decoding for animacy. Mean classifier performance (*d’*) for both the categorization task (red) and distracted viewing task data (orange) plotted over time. Shaded regions above and below the mean lines indicate +/- 1 *SEM* across subjects. Color-coded asterisks indicate time points at which classifier performance was significantly above chance, using a Wilcoxon signed rank test (* = false discovery rate (FDR) adjusted *p* < 0.05). Decoding onset for both data sets (60ms) is indicated by a dashed vertical line. The period of peak decoding is indicated by a gray box extending 120–240 ms post-stimulus onset. The bar along the x-axis indicates the stimulus duration.

We first replicated previous studies showing that animate vs. inanimate object exemplars can be decoded on a trial-by-trial basis from the MEG time-series data [7–8]. Linear discriminate analysis (LDA; [20]) was used at each time point to classify the category of the exemplar (animate or inanimate) displayed to the subject. Fig 3 shows decoding performance as a function of time for the MEG data from the categorization and distracted viewing tasks epoched from -100 to 600 ms post-stimulus onset (reported in *d’*). Performance was similar for both data sets. The decoding onset (i.e. the first time point at which decoding is above chance) was 60 ms post-stimulus for both tasks. Following the onset, there was a broad peak in decoding with two smaller peaks. The first smaller peak at 140 ms was the same for both task data sets. The second smaller peak occurred at 220 and 240 ms for the distracted viewing and categorization task respectively. Our main theoretical interest was the time of peak decoding–the optimal time for decision information to be “read out” from the brain’s representation of the stimulus. To define this time-period, we calculated the mean decoding performance for the two smaller of peaks (*d’* = .61), and then tested all time points to determine those that were not significantly different from this mean peak value (see Methods). The time points from 120–240 ms were not significantly different from the peak value. In what follows, we will refer to this period as the *period of peak decoding*, which is highlighted as a grey region in Fig 3.

### Representational distance predicts reaction times for categorization throughout the decoding time-course

We sought to test whether distance from a decision boundary through an activation space constructed at the period of peak decoding would predict RTs for categorization. For each time point we constructed a multidimensional activation space from the MEG sensor data (see Methods). In the activation space, the average activity patterns of individual exemplar representations were the exemplars’ coordinates. LDA was used to calculate a discriminate axis and decision boundary for animacy through this space. The individual exemplar activation patterns were then projected onto the discriminate axis, and we computed the distance of the exemplar pattern from the decision boundary (see Fig 1A and 1B). The procedure was done separately for the categorization and distracted viewing task MEG data sets, yielding “representational distances” (one for each object exemplar) for each task at each time point.

**Fig 4:**
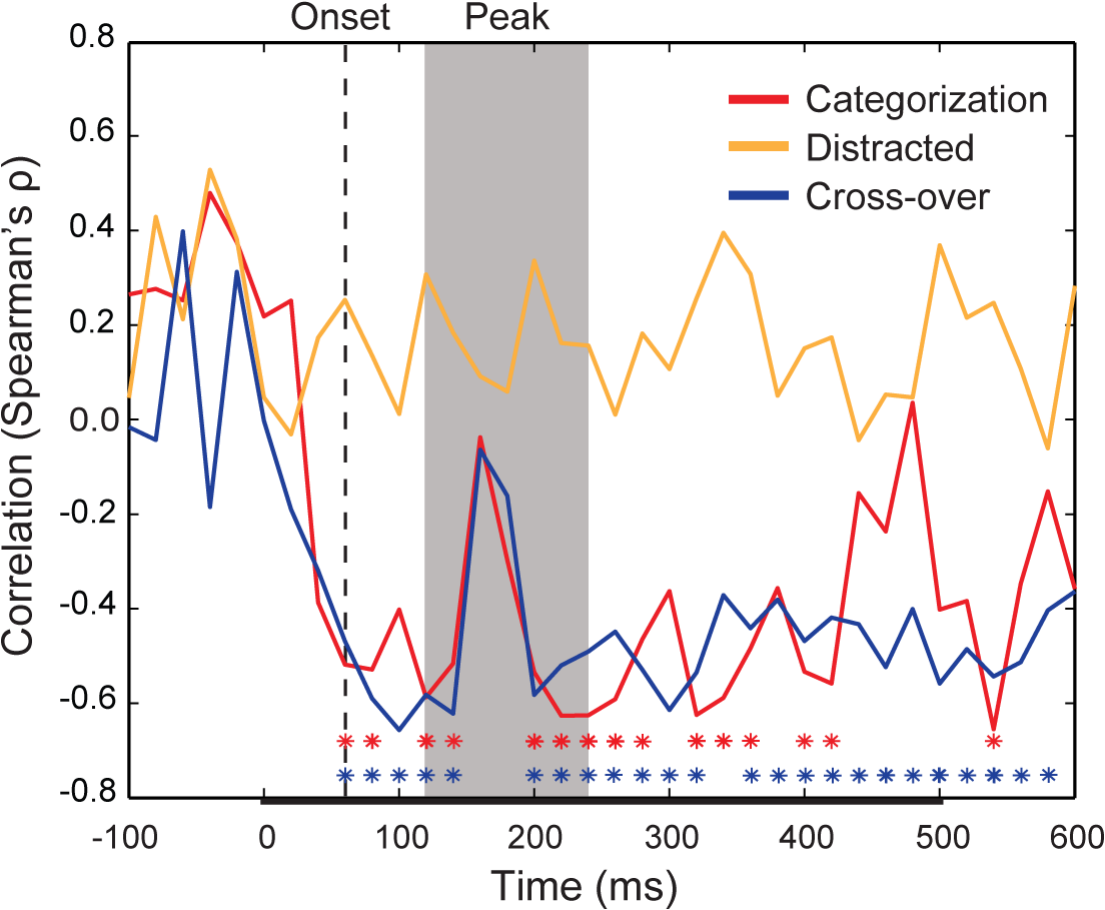
Time-series correlation between representational distances from the animacy boundary and categorization task reaction times. The time-varying rank-order correlation (Spearman’s ρ) between the average object exemplar representational distance and average reaction time across subjects for the categorization task (red), distracted viewing task (orange), and cross-over between the tasks (blue), in which representational distances from the distracted viewing task were correlated with the median RTs for the categorisation task. Color-coded asterisks indicate time points at which a correlation between distance and RT achieves significance (* = FDR adjusted *p* < 0.05). The decoding onset is indicated by dashed vertical line (60 ms). The period of peak decoding is indicated by the gray shaded region extending from 120–240 ms post-stimulus onset. The bar along the x-axis indicates the stimulus duration.

To study the relationship between RT behaviour and the emerging representation of the stimuli in the brain, the time varying distances of the object exemplars from the representational boundary for animacy were correlated with RT performance on the categorization task. To reduce noise and increase statistical power, we performed a fixed-effect analysis, utilizing the average representational distances at each time-point and normalized median RTs across subjects. Based on our primary hypothesis, we predicted that RTs for the categorization task would correlate negatively with representational distances during the period of peak decoding. Contrary to our expectations, the correlation time-course was significant at multiple time-points from decoding onset onwards (Fig 4). Several of these time points were before (< 120 ms) or after (> 240 ms) peak decoding, while the correlation notably failed to achieve significance in the middle of the period of peak decoding (160–180 ms).

We next reasoned that median RTs for the distracted viewing task should *not* correlate with distances from an animacy boundary, since there is no reason to believe that distance from such a boundary should be predictive for categorizing letters as vowels or consonants. To test this null prediction, we grouped RTs for the distracted viewing task based on object exemplar, so that decision time reflected the time for observers to categorize the co-occuring letters as vowels and consonants. Based on such a grouping, there should be no relationship between median RTs and distance from the animacy boundary. As expected, at no time point did the correlation between distractor task RTs and distances from the animacy boundary achieve significance (Fig 4).

Carlson et al. [14] correlated representational distances from IT, measured using fMRI while subjects passively viewed object exemplars, with RTs for object categorization, which were obtained from a separate group of subjects. Even though the fMRI subjects were not performing a categorization task, representational distances still correlated with RTs. The findings of Carlson et al. suggest that the structure of neural representations in the visual system driving categorization behaviour is not wholly task-dependent. We reasoned that even though subjects “passively” viewed exemplars while performing the distracted viewing task the representational distances constructed from the distracted viewing task MEG data would still be predictive of RTs for the “active” categorization task. We measured the correlation between representational distances for the distracted viewing task with the median RTs for the categorization task. This *cross-over* correlation followed a similar trajectory to the categorization task correlation time-course (Fig 4), achieving significance for nearly the complete time-course from decoding onset onwards, and again with the notable exception of 160–180 ms during the period of peak decoding. These findings show that the emerging representation that predicts RTs is a core representation that is constructed by the visual system even during passive viewing, and thus must be independent of task-specific decision or motor processing (see Methods).

**Fig 5:**
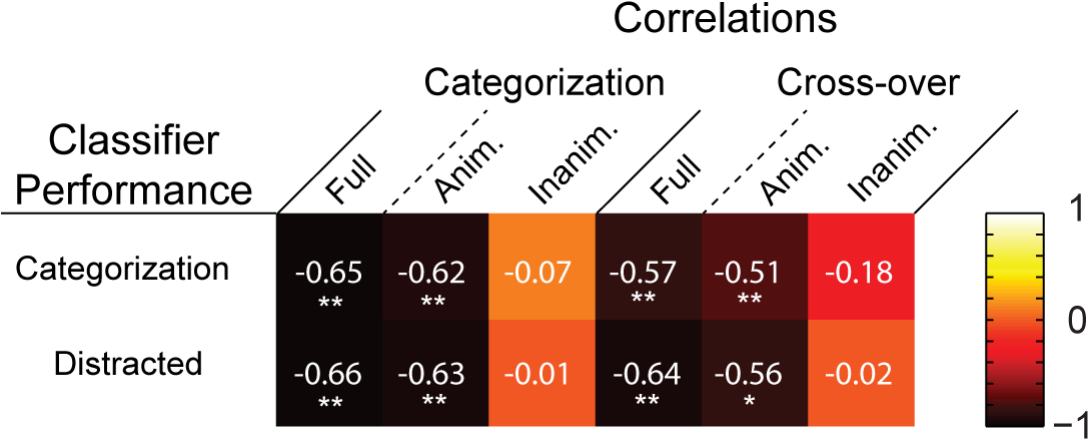
Matrix of correlations between decoding time-courses and representational distance-RT correlation time-courses. The “heat” of each tile reflects the strength of the rank-order correlation (Spearman’s ρ) between a decoding time-course and a representational distance-RT correlation time-course. Asterisks indicate significant correlations (* = *p* < .01; ** = *p* < .001).

In order to quantify the relationship between decoding and the representational distance-RT correlations, we correlated both decoding time-courses with both significant representational distance-RT correlation time-courses. We observed a significant negative correlation between each pair of decoding and correlation time-courses (Fig 5), reflecting the fact that as classifier performance increased there was an increase in the negative correlation between representational distance and RTs. Thus the relationship between representational distance and RTs appears to track the time-course of decoding.

### Animate exemplars drive relationship between representational distance and reaction times for categorization

There is evidence that animate and inanimate objects are represented differently in the ventral visual stream of humans and monkeys [13, 21–22]. In their study, Carlson et al. [14] found that the correlation between distance and RTs that they observed was driven entirely by animate exemplars, with no significant correlation between representational distance and RTs for inanimate exemplar stimuli. Thus we sought to determine whether the correlations we observed between representational distance and RTs were also driven by the data for the animate exemplars.

**Fig 6:**
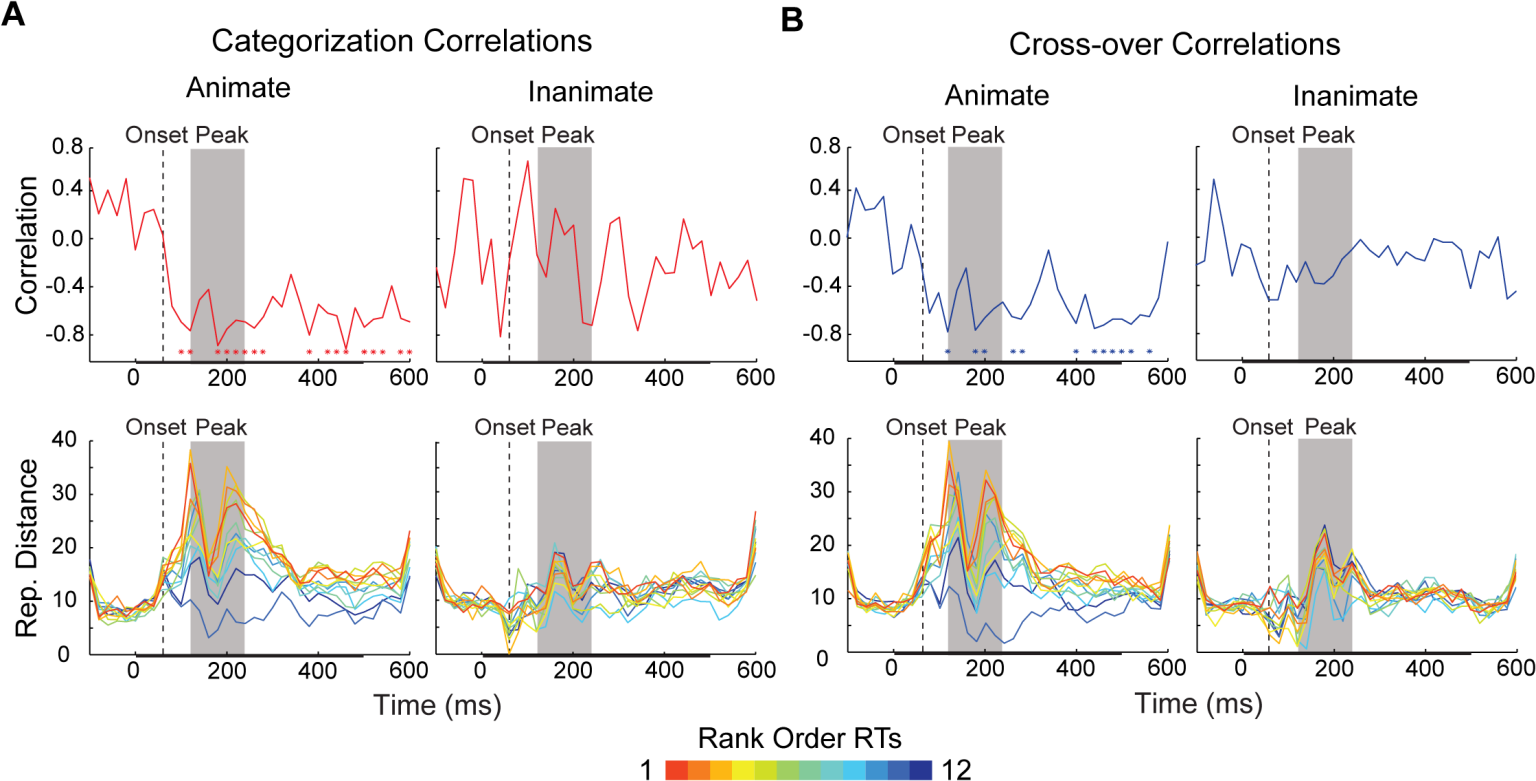
Time varying representational distance for individual exemplars separated by object category. Time varying (A) categorization task (red) and (B) cross-over (blue) rank-order correlations (Spearman’s ρ) for animate and inanimate exemplar stimuli. The color-coded asterisks in the top row of plots indicate time points at which there is a significant correlation between distances and RTs (* = FDR adjusted *p* < 0.05). The bottom row of plots displays the distances from animacy decision boundaries at each time point, computed for both the categorization task and distractor task data sets. Each time-varying line depicts the representational distance for an individual exemplar stimulus, at each 20 ms time point (either animate or inanimate). The color of each line is based on the rank-order of the median RT for each exemplar (rank is always within category). In all plots, decoding onset is indicated by dashed vertical line (60 ms). The period of peak decoding is indicated by a gray box extending 120–240 ms post-stimulus onset. The bar along the x-axis indicates the stimulus duration.

**Fig 7:**
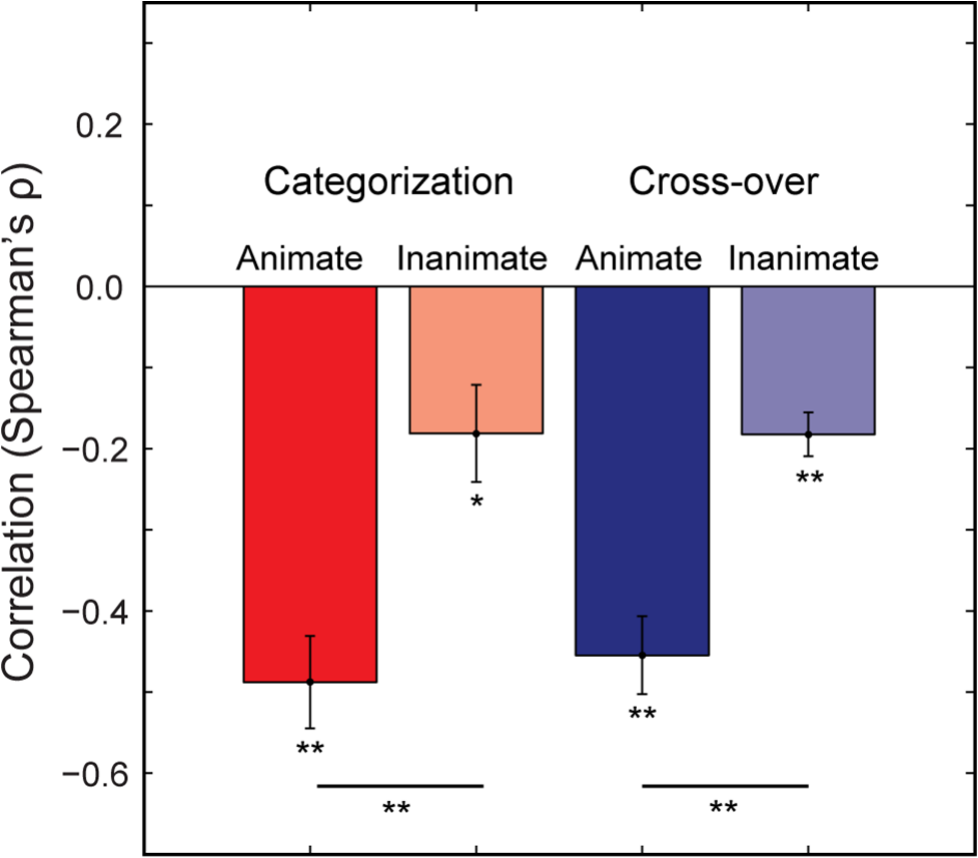
Time-averaged (0–600 ms) correlations between representational distance and RTs for both animate and inanimate object exemplars. Mean time-averaged categorization (red) and cross-over (blue) correlations (Spearman’s ρ) between representational distances and RTs. Asterisks indicate significant comparisons (Wilcoxon Signed Rank Test; * = *p* < .01; ** = *p* < .001). Error bars indicate +/- 1 *SEM* across time-points.

We again measured the categorization task and cross-over correlation time-courses, this time analysing the data separately for animate and inanimate objects (Fig 6A and 6B). The animate correlations showed a consistent negative pattern, achieving significance around the period of peak decoding, and at later time-points (≥ 380 ms). When time-averaged (0–600 ms), the animacy correlations were significantly greater than their inanimate counterparts, though the latter correlations were also significant (Fig 7). However, only the animate exemplar correlation time-courses were significantly correlated with the decoding time-courses (Fig 5). Thus it appears that the animate examplars are indeed driving the time-varying relationship between representational distances and RTs. Furthermore, given that the mean RT for the categorization task was 469 ms, the significant time points after the period of peak decoding likely reflect the continued representation of animate exemplars after a decision was already made by observers (or would have been made, in the case of the cross-over correlation). Thus our primary prediction is borne out by the correlation time-courses for animate exemplars: information about (animate) object categories is being read-out during the period of peak decoding (Fig 6).

The lower plots of Fig 6A and 6B show the averaged representational distances for animate and inanimate stimuli, color-coded by the rank-order categorization task RTs. Qualitatively, the representational distances for animate exemplars appear more separable than those for inanimate exemplars, increasing in relative distance considerably around the time of peak decoding. Notably, the distances for animate exemplars collapse at 160–180 ms, which corresponds to a slight dip, or “valley”, during the period of peak decoding (Fig 3), and is the point when the correlation time-courses for mean representational distance and median RT lose significance (Fig 4). This “peak and valley” pattern in classifier performance is also found in the results of other studies that have decoded exemplars based on animacy [7–8]. The collapse in distances at 160–180 ms appears to explain why classifier performance remains high at these time points, but the correlation time-courses are not significant: at that time there is substantial relative distance between category (as reflected in distance from the decision boundary, or 0 on the y-axes), but minimal relative distance within category, resulting in a poorer ordering with respect to rank RTs.

### Latency and amplitude of sensory peaks do not predict reaction times for categorization

**Fig 8:**
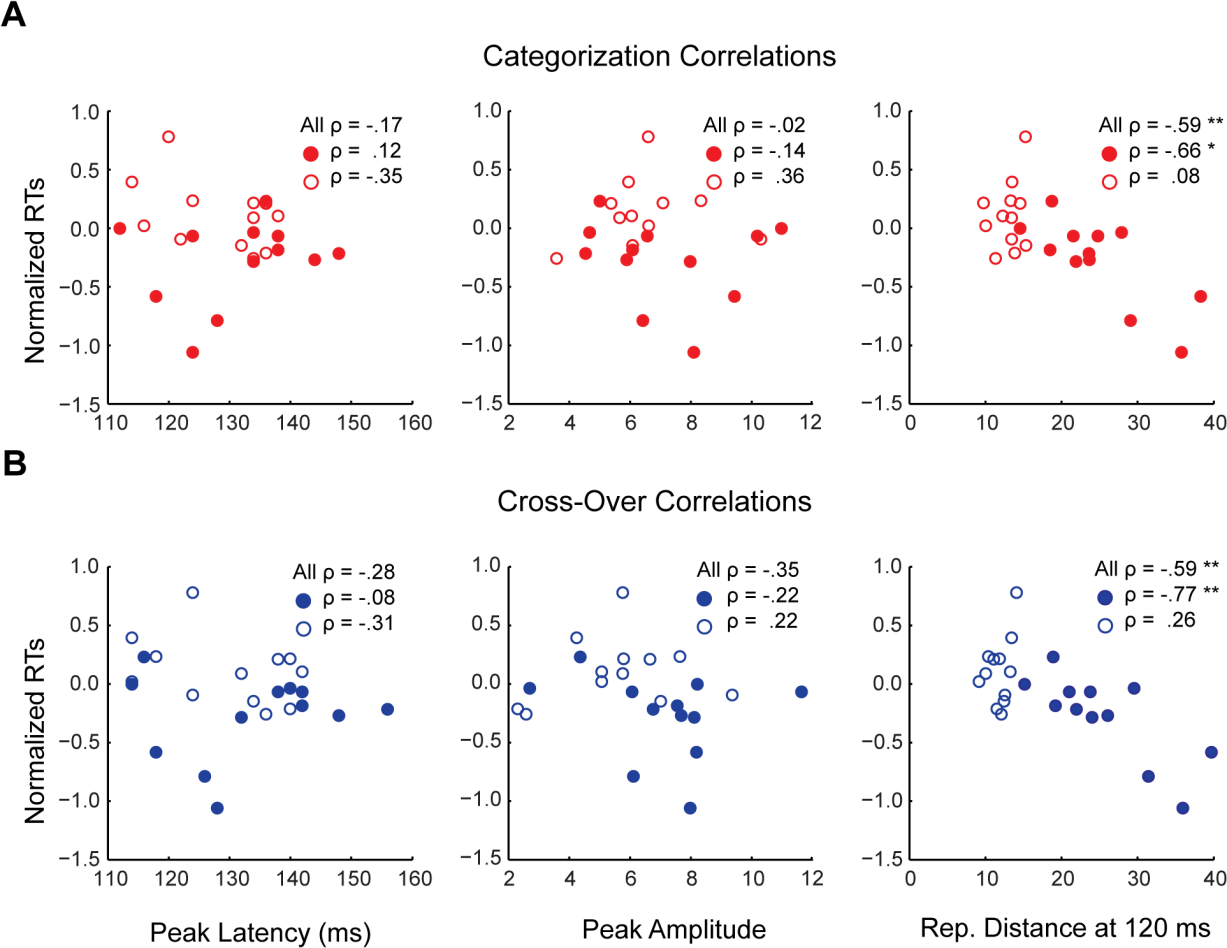
Correlations between sensory peak latencies and amplitudes and categorization task RTs. Scatter plots for the rank-order (A) categorization and (B) cross-over correlations (Spearman’s *ρ*) between peak latency and peak amplitude and median normalized RTs for the categorization task. (A) Red circles indicate animate exemplar data points, while red rings indicate inanimate data points. (B) Blue circles indicate animate exemplar data points, while blue rings indicate inanimate data points. Peak amplitude scale is in units of 10^−14^ T. For comparison also plotted are the correlations between representational distance and RTs at 120 ms. Asterisks indicate significant correlations (* = *p* < .01; ** = *p* < .001).

One question is whether the observed relationship between representational distance and RTs might track evoked responses observable using more conventional analyses. For example, Philiastides and Sajda [23] found that large amplitudes in difference waveforms coincided with their best decoding time-windows. Similarly, in our data differences in the amplitude of the evoked responses for animate and inanimate exemplars are evident in the grand average scalp topographies at the decoding peaks (S1 Fig). Furthermore, difference waveforms were significant both at peak decoding, and at ~ 380 ms onward (S2 Fig). Thus we asked whether the timing or magnitude of evoked responses might also predict RTs for categorization. To address this question, we isolated the peak latency and amplitude of early sensory peaks (< 160 ms post-stimulus onset) for each individual exemplar from the grand averaged categorization and distracted viewing task MEG data. We then correlated these peak latencies and amplitudes with the median normalized RTs. If RTs reflect peak latency, we expect a positive correlation (greater latency predicting slower RTs), while if RTs reflect peak amplitude, we expect a negative correlation (greater amplitude predicting faster RTs). Neither correlation was significant, for either the categorization task or distracted viewing task peaks, even when separated by animacy (Fig 8). For comparison with the lower plots of Fig 6, S3 Fig also depicts the sensory peaks for each exemplar, separated by animacy, and color-coded for rank-order RT. As can be seen in the plots, the peak waveforms exhibit no clear ordering with respect to rank RTs. Given these null results, the observed relationship between representational distance and RTs does not appear to straightforwardly track properties of sensory peaks in the MEG evoked response.

## Discussion

In the present study we showed when and how the emerging representation of objects in the brain, as revealed using MEG decoding, can be related to categorization behaviour. Following previous work with fMRI showing that representational distance from a category boundary in the activation space of human IT predicts RTs for object categorization [14], we sought to determine at what time representational distances predicted RT behaviour. The period of peak decoding provides the optimal period at which to read-out information about stimulus category. Thus we hypothesized that representational distances and RTs would exhibit the same relationship during this period, and predicted that RTs would negatively correlate with distance from a decision boundary through the activation spaces at the time of peak classifier performance. We found that representational distances and RTs correlated at most time-points following decoding onset. It was only when the data was separated by animacy that our prediction was supported: RTs began to negatively correlate with representational distance during the period of peak decoding, with the time-varying correlation tracking the time-course of decoding more generally. Our results have implications for (i) research on ultra-rapid object categorization, (ii) how time-resolved decoding results are interpreted to make inferences about neural representations, as well as for (iii) hypotheses concerning the possible neural loci of perceptual decision-making in vision.

### Implications for ultra-rapid categorization

The fact that ultra-rapid categorization can be performed with some reliability suggests that information about stimulus category is available shortly after stimulus presentation. Congruent with this conjecture, information about object category can be decoded 60–100 ms after stimulus onset. However the availability of such information at such early latencies does not show, by itself, that this information is being used by subjects in a task related manner. In the present study, classifiers trained on the categorization task and distractor task data both achieved significant above chance performance as soon as 60 ms after stimulus onset, in line with previous MEG decoding experiments [7–8, 24]. When data was pooled across subjects, the first time point at which we observed a significant negative correlation between RTs and distance from an animacy boundary was decoding onset. Such an early latency for the predicted relationship between distance and RTs is consistent with existing psychophysical research showing reliable saccadic RTs at very short latencies of less than 200 ms [3, 5]. An enticing prospect is that future research might use the present distance approach to MEG decoding, in conjunction with behavioural measures, to further characterize the temporal dynamics of ultra-rapid object categorization. For example, there is evidence that RTs for superordinate categories (e.g. animal or non-animal) can be significantly faster than those for subordinate categories (e.g. dog or cat) [25–26]. In contrast, while MEG decoding onset does not appear to vary with object category, classifier performance peaks earlier for subordinate relative to superordinate categories [7–8]. Future research could use our distance approach to reconcile the apparent tension between these behavioural and decoding results.

### Implications for the interpretation of time-resolved decoding

It has been claimed that time-resolved decoding methods reveal the temporal dynamics of “representational geometries” in the brain [11, 27]. However significant above chance classifier performance only warrants the inference that some information about a stimulus or task condition is available, but not that it is being used by the brain [28]. In this respect, the dynamics of the correlations between distance and RTs that we report are notable. The few MEG decoding results to date consistently show a “peak and valley” topography when a classifier is used to discriminate between animate and inanimate exemplars, as well as between other categories. From decoding onset classifier performance climbs to an initial peak, followed by a dip into a high altitude valley, before rising to a second peak. This can be seen in Carlson et al. ([7], Fig 3E), and Cichy, Pantazis and Oliva ([8], Fig 2A), as well as the present results (Fig 3). It is noteworthy that the “valley” in decoding performance aligns with an approximate point, 160–180 ms, at which there is a collapse in the representational distances (Fig 6A and 6B), and a corresponding lack of correlation between distance and RTs (Figs 4, 6A and 6B). We have highlighted this qualitative feature of our results because it suggests that although a classifier is able to use information latent in the patterns of neural activity at this time, the brain itself might not be using this information. Decodability reflects classifier performance, while the correlation between representational distance and RTs reflects human performance, and so the latter likely provides a better proxy of when in time object representations have emerged, or been “untangled” [29]. At the same time, the fact that the correlations between distance and RTs closely track classifier performance (Fig 5) provides some evidence for the idea that the brain is indeed a decoder of its own neural signals [11].

### Implications for neural models of perceptual-decision making

While our study shows that representational distance predicts RT behaviour, one question is how to connect this spatial measure, distance, to the temporal dynamics of the decision process. Such a connection can be established using sequential analysis models, which have used to uncover the neural loci of perceptual-decision making [30, 31]. These models have been related to neural activity, in humans and monkeys, using a variety of stimuli, tasks, and recording methods, including cellular recordings, fMRI, and EEG/MEG [32–38]. While varying in their details, all sequential analysis models characterize differences in RT as resulting from variation in the evidence accumulation (or “drift”) rate toward a decision threshold. In order to make a connection between the evidence accumulation process and the brain’s population codes (as reflected in the patterns of neural activity clustered in high-dimensional activation spaces), Carlson et al. [14] implemented a simple sequential analysis model to show that distance from a decision boundary in human IT results in differences in accumulation rate to a decision threshold. If drift rate is the only free parameter of the model, it can be fixed based on distance, since the starting point of the accumulation process is in effect equivalent to the decision boundary as specified by signal detection theory ([39–40]). When fixed in this way, drift rate scales with distance from a decision boundary: shorter distances entail slower drift rates, and longer distances entail faster drift rates. Thus, sequential analysis models, a prominent theoretical fixture in the decision-making literature, provide a bridge from representational distances to the temporal dynamics of the decision processes.

We did not apply a sequential analysis model to our data. However, as discussed by Carlson et al. [14], it follows analytically from the observed correlations between distance and RTs that such an application is possible. For example, with our data time averaged distances during peak decoding could be used to set the drift rate parameter for each individual object exemplar, resulting in a positive correlation between drift rate and RTs (cf. [41]). In so far as the present experiment provides further evidence that representational distance predicts RTs, our results supports the hypothesis that representational distance determines the quality of evidence that feeds into the evidence accumulation process. Although the correlations we report likely reflects multiple brain areas rather than a single decision variable accumulating over time, we do believe our results also offers an important perspective on how decision variables are implemented in the brain; namely, as a trajectory through high-dimensional activation spaces reflecting the transformation of information over time [29,39]. More broadly our findings support the idea that representing is partially constitutive of the decision process for categorization [14].

### Summary

Motivated by recent findings, we sought to determine how the time-varying signal for objects, as identified by time-resolved decoding methods, can be related to behaviour. Previous research has shown that distance from a partitioning through a high-dimensional activation space can be used to predict RTs. We reasoned that since peak-decoding indexes the optimal time for reading-out information regarding stimulus category, it would be during the period of peak decoding that we would witness a relationship between distance and decision. In line with our expectations, RTs negatively correlated with distance from decision boundaries during the period of peak decodability, but only when our data was separated by object category. Our results provide evidence that the time course of decoding indeed reflects the emergence of representations for objects in the visual system. Furthermore, they also give credence to the thesis that representing and deciding do not necessarily reflect a clean partitioning between sensory evidence and its evaluation, but are instead fused during the process of categorization.

## Methods

### Ethics statement

The research in this study was approved by the Institutional Review Board at the University of Maryland, College Park.

### Participants

Thirty subjects from the University of Maryland, College Park, participated in the experiment (15 female; mean age = 21.1). All subjects had normal or correct to normal vision and were compensated financially for participating. The MEG data of one subject was corrupted, so only the data of the remaining 29 subjects was analysed.

### Stimuli and tasks

Stimuli were twenty-four segmented natural images of objects, consisting of a heterogeneous mix of animate and inanimate exemplars (12 animate, 12 inanimate): human and animal faces and bodies, and artificial and natural objects. Experiments were run on a Dell desktop PC computer running Matlab (Natick, MA). Stimuli were displayed on a translucent screen located 30 cm above participants in the MEG chamber. On the display, the stimuli were approximately 4 degrees of visual angle. Superimposed onto each image was a small fixation circle (0.4 degrees of visual angle) containing a letters drawn from the following set of vowels and consonants: vowels = ‘A’, ‘E’, ‘I’, ‘O’,’U’; consonants = ‘R’, ‘N’, ‘X’, ‘S’, ‘G’.

Subjects performed one of two tasks on alternating runs of the experiment (Fig 2). The *categorization* task required subjects to respond whether the exemplar was animate or inanimate (i.e. whether it was “capable of moving on its own volition”). The *distracted viewing* task required subjects to respond whether the character in the fixation circle was a vowel or consonant. Subjects were instructed to respond as quickly and accurately as possible while performing the tasks.

To remove any potential confounds associated with motor activity, the mapping between object category/letter type and response alternated on each run of each task. For example, if on the first categorization task run subjects responded with the left button for animate stimuli, and right button for inanimate stimuli, then on the next categorization task run the mapping would be reversed. Subject’s choice and RT data were collected for each trial. After responding, subjects were given feedback on their performance: if subjects responded correctly the fixation circle flashed green; if the subjects responded incorrectly, or failed to respond during the response period, then the fixation circle would flash red (Fig 2).

Trials were structured as follows (Fig 2). Each stimulus was presented for 500 ms, and subjects had 1000 ms (including the stimulus duration) to respond. The inter-stimulus interval for each trial was randomly selected from the range 900–1200 ms. Each image was presented 8 times in each run, in random order, resulting in 192 trials per run. Subjects performed 8 runs, with 4 categorization runs, and 4 distractor runs, resulting in 768 trials per task. At the end of each run subjects were provided feedback on their response accuracy for the run (percentage correct).

### MEG data acquisition and preprocessing

The neuromagnetic signal of the subjects was recorded using a 160 channel (157 recording; 3 reference) whole-head axial gradiometer (KIT, Kanazawa, Japan). Signals were digitized at 1000Hz, and filtered online from 0.1 to 200 Hz using first-order RC filters. Offline, time-shifted principle component analysis (TSPCA) was used to denoise the data [42]. TSPCA filters the data using the reference channels to estimate environmental noise. After denoising the data, trials were epoched from 100 ms pre-stimulus to 600 ms post-stimulus. Eye-movement artefacts were removed using an automated algorithm in Matlab. The average rejection rate of trials due to eye-movements was 2.1% with 2.6% *SD* across subjects.

For the MEG decoding analyses, PCA with a threshold of retaining 99% of the variance was used to reduce the dimensionality of the datasets. The sampling rate was reduced to 50Hz to increase signal to noise, resulting in 36 time-points with a 20 ms resolution. The data was downsampled using the decimate function in Matlab, which first applies a low-pass Chebyshev Type I filter. Filtering when downsampling introduces a latency offset (estimated by simulation to be 20 ms), which was corrected for after downsampling. For the conventional time-series analyses, the data was downsampled to 500 Hz and low-pass filtered (Butterworth) at 40 Hz offline using SPM8 ([43]), resulting in time-points with a 2 ms resolution and negligible latency offset. No off-line high-pass filtering was applied to the data for either the decoding or conventional analyses.

### Sliding time-window decoding analysis

The decoding analysis was run separately for the categorization task and distracted viewing task MEG datasets. For each set of data, we used a naïve Bayes implementation of linear discriminate analysis (LDA, [20]) to perform single-trial classification of animate and inanimate objects from the scalp topography at each time point. Generalization of the classifier was evaluate using k-fold cross validation with a 9:1 training to test ratio. In this procedure, the neuromagnetic data for all trials of a task were randomly assigned to 10 bins approximately equal in size. Nine of the bins were pooled to train the classifier, and the trials in the remaining bin were used to test the classifier. This procedure was repeated 10 times such that each trial was tested on exactly once. The decoding analysis was run on each time point to measure the time varying decoding performance for animacy. Classifier performance is reported in terms of *d’*. Mean classifier performance at each time-point was tested for significance using the Wilcoxon signed rank test. To correct for multiple comparisons we also computed the false discovery rate (FDR) adjusted *p*-values with α = 0.05. The same correction was performed for all other tests of statistical significance that involved multiple comparisons (i.e. testing at each time point).

To quantify the period of peak decoding, we first calculated the mean value of the two peaks in classifier performance for the categorization task and distractor task data (*d’* = .61). We then tested whether classifier performance at each time-point was significantly different from this peak value using the Wilcoxon signed rank test (FDR adjusted *p* < .05). The period of peak decoding was defined as all time-points at which at least one classifier time-course was not significantly different than the peak performance value.

### Distances in activation space

An activation space is an *N*-dimensional space with the number of dimensions determined by the number of components retained after dimensionality reduction using PCA (see above). LDA was used to compute a discriminant axis for animacy in the activation space constructed for each 20 ms time-point. The mean activation pattern for each exemplar was projected onto the discriminant axis. A naïve Bayes classifier was then used to compute a decision boundary for animacy for each MEG dataset. Individual exemplar distances were computed as the absolute value of the Euclidean distance of a pattern of activity for an exemplar from the decision boundary. The exemplar distances were computed at each time point providing a time varying measure of the distance of object exemplar representations from the decision boundary. The partitioning of the activation space generated by LDA is not perfect. Although an exemplar is animate, a pattern of activity for the exemplar might fall on the inanimate side of the partition, since LDA and the classifier only provide the best (not perfect) linear partitioning of a space. To ensure that each time point had equal data for computing the correlations, classification accuracy was not taken into consideration when computing the distances. The above procedure was carried out separately for the two data sets, yielding a set of categorization task distances for each time-point, and a set of distracted viewing task distances for each time-point.

### Correlating distances in activation space with reaction times

RTs from the categorization and distracted viewing tasks were analysed after individual subjects’ RT were normalized and pooled. We measured the rank-order (Spearman’s *ρ*) correlations between the normalized median RTs and the representational distances at each of the 36 time points. We measured three different correlations between distance and RTs: (i) the correlation between object categorization task RTs and distances computed from the categorization task time-series data (the *categorization* correlation); (ii) the correlation between distracted viewing task RTs pooled by object exemplar and distances computed from the distracted viewing task time-series data, and (iii) the correlation between the categorization task RTs and the distances computed from the distractor task time-series data (the *cross-over* correlation). We also measured the categorization and cross-over correlations separately for animate and inanimate exemplars. These comparisons produced correlation time-courses which were also correlated (Spearman’s *ρ*) with the decoding time-courses, or time-averaged.

### Analysis of difference waveforms and sensory peaks

To determine whether a substantial difference between evoked responses for animate and inanimate exemplars might coincide with the period of peak decoding, we generated difference waveforms (animate - inanimate) from both the categorization task and distracted viewing task MEG data. First, using the grand averaged data, we isolated the five sensors that showed the largest (positive) amplitude in the difference waveforms at the same peak times that were used to define the period of peak decoding (categorization task: 140 and 240 ms; distracted viewing task: 140 and 220 ms). The approximate location of these sensors can be seen in S1 Fig. This resulted in four sets of five sensors, one for each local decoding peak. Second, we averaged the data from each set of five sensors for each individual subject, and tested for significance at each time-point using the Wilcoxon signed rank test (correcting for multiple comparisons using FDR).

To test whether sensory peak latencies or amplitudes might predict RTs, we first isolated the five sensors that showed the largest (positive) amplitude -100–160 ms post stimulus onset for animate exemplars, using the grand averaged data. The approximate location of these sensors can be seen in S1 Fig. Next, again using the grand averaged data, we averaged the data from these five sensors for each individual exemplar, and calculated the latency and amplitude of the maximum peak -100–160 ms. The sensory peaks isolated by this procedure are depicted in S3 Fig. Finally, we then measure the rank-order correlations (Spearman’s *ρ*) between the peak latencies and amplitudes and median normalized RTs.

All channel selection for these analyses was done using an automated search for the maximum amplitude of the evoked responses within the pre-defined time-windows.

## Supporting Information

S1 Fig Grand averaged scalp topographies at peak decoding. Plots show the grand average scalp topographies for animate and inanimate exemplars, as well as their difference (animate - inanimate), at each local decoding peak. Black dots indicate the sensors selected for isolating data for further analysis (see: Results; Methods; and Figs 8, S2 and S3). All amplitude scales are in units of 10^−14^ T.
(TIF)

S2 Fig Difference waveforms with maximum amplitudes at decoding peaks. Each waveform depicts the grand average data from five sensors that had maximum amplitude at the local decoding peaks. Lighter colored waveforms have maximum amplitude at the first peak (140 ms), while darker colored waveforms have maximum amplitude at the second peak (categorization task: 240 ms; distracted viewing task: 220 ms). All response amplitude scales are in units of 10^−14^ T. Color-coded asterisks indicate time points at which the amplitude of the difference waveforms achieved significance based on a Wilcoxon signed rank test (* = FDR adjusted *p* < 0.05). The decoding onset is indicated by dashed vertical line (60 ms). The period of peak decoding is indicated by the gray shaded region extending from 120–240 ms post-stimulus onset. The bar along the x-axis indicates the stimulus duration.
(TIF)

S3 Fig Sensory peak waveforms for object exemplars. Each waveform depicts the grand averaged data for an individual exemplar, from five sensors with maximum amplitude for animate exemplars -100–160 ms post-stimulus onset. Each plot contains the waveform for animate or inanimate exemplars, from the categorization task or distracted viewing task MEG data. The color of each waveform is based on the rank-order of the median normalized RT for each exemplar (rank is always within category). All response amplitude scales are in units of 10^−14^ T. The decoding onset is indicated by dashed vertical line (60 ms). The period of peak decoding is indicated by the gray shaded region extending from 120–240 ms post-stimulus onset. The bar along the x-axis indicates the stimulus duration.
(TIF)
