# Supplementary figures and images for: Emerging Object Representations in the Visual System Predict Reaction Times for Categorization
Source: PLoS Comput Biol. 2015 Jun 24;11(6):e1004316. doi: 10.1371/journal.pcbi.1004316 (PMC4479505; doi:10.1371/journal.pcbi.1004316)

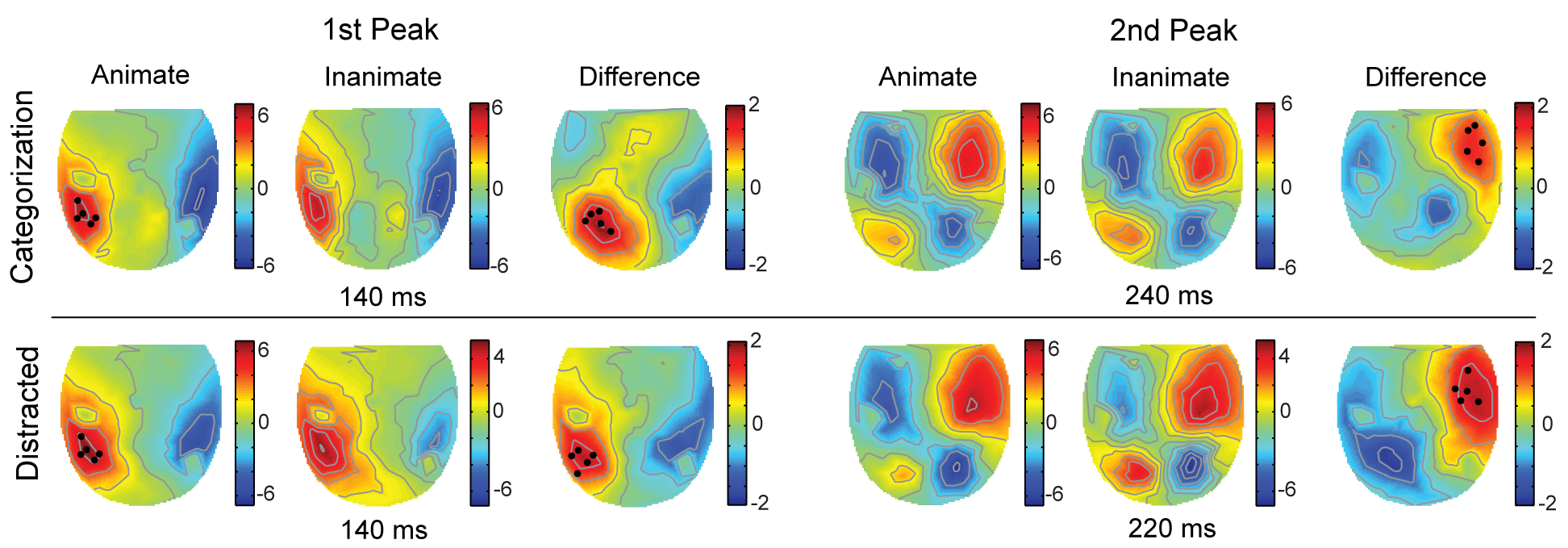

Supplement: S1 Fig — Plots show the grand average scalp topographies for animate and inanimate exemplars, as well as their difference (animate - inanimate), at each local decoding peak. Black dots indicate the sensors selected for isolating data for further analysis (see: Results; Methods; and Figs 8, S2 and S3). All amplitude scales are in units of 10−14 T. (TIF) [file pcbi.1004316.s001.tif]

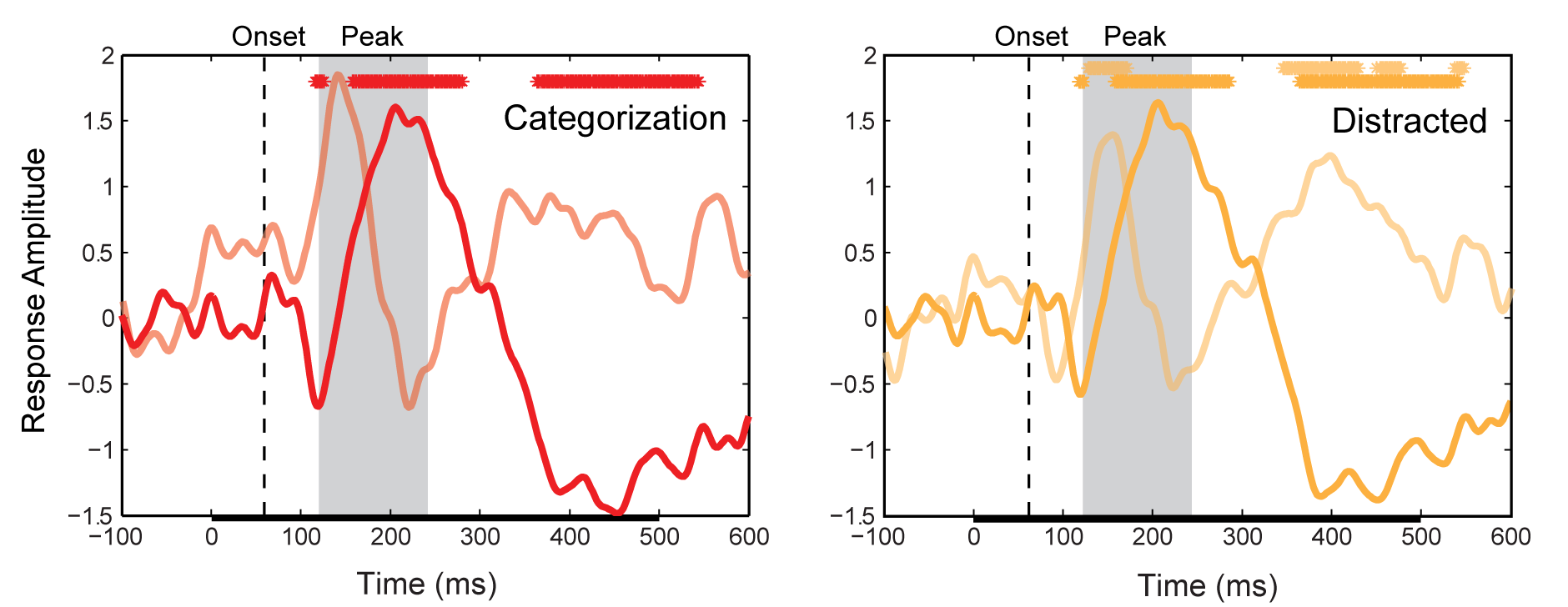

Supplement: S2 Fig — Each waveform depicts the grand average data from five sensors that had maximum amplitude at the local decoding peaks. Lighter colored waveforms have maximum amplitude at the first peak (140 ms), while darker colored waveforms have maximum amplitude at the second peak (categorization task: 240 ms; distracted viewing task: 220 ms). All response amplitude scales are in units of 10−14 T. Color-coded asterisks indicate time points at which the amplitude of the difference waveforms achieved significance based on a Wilcoxon signed rank test (* = FDR adjusted p < 0.05). The decoding onset is indicated by dashed vertical line (60 ms). The period of peak decoding is indicated by the gray shaded region extending from 120–240 ms post-stimulus onset. The bar along the x-axis indicates the stimulus duration. (TIF) [file pcbi.1004316.s002.tif]

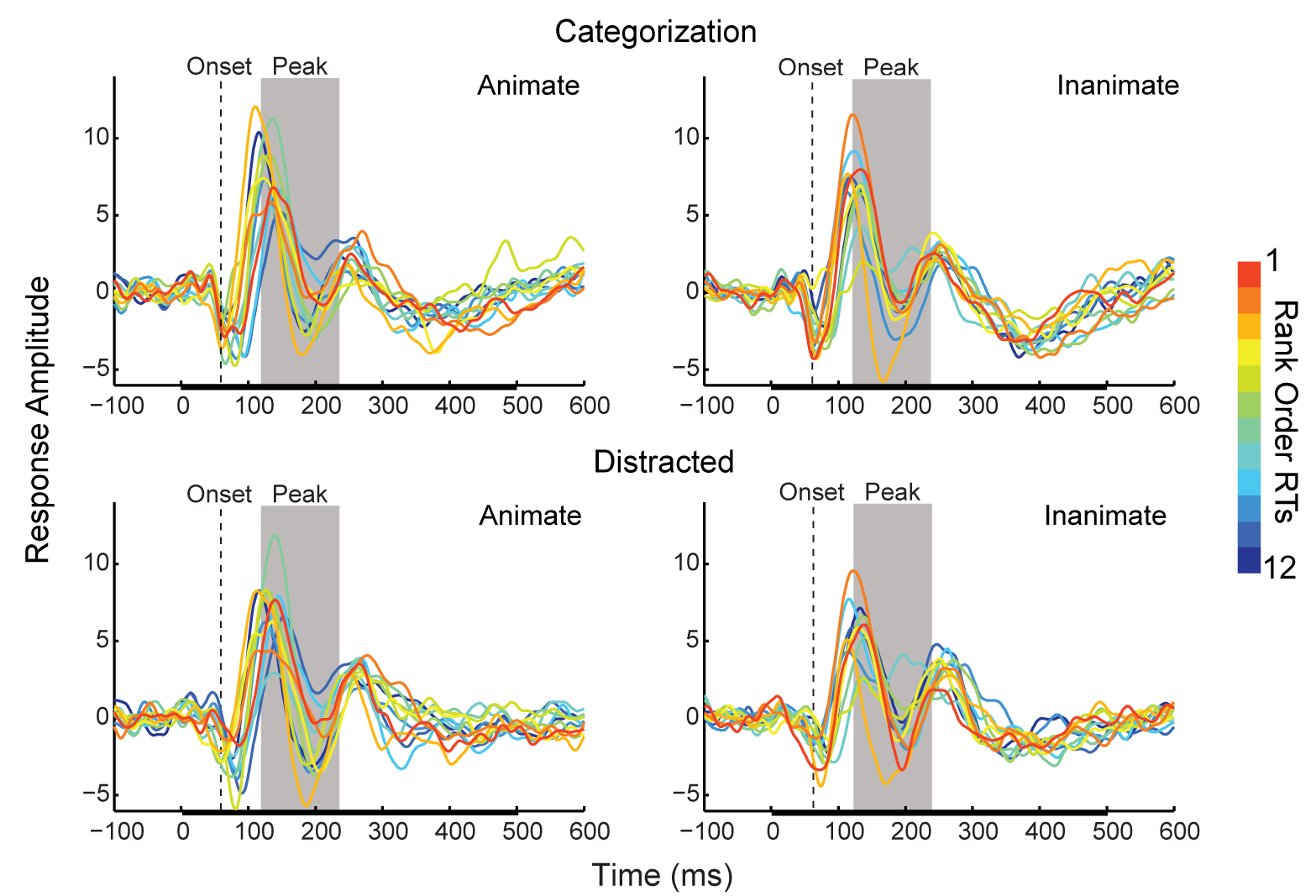

Supplement: S3 Fig — Each waveform depicts the grand averaged data for an individual exemplar, from five sensors with maximum amplitude for animate exemplars -100–160 ms post-stimulus onset. Each plot contains the waveform for animate or inanimate exemplars, from the categorization task or distracted viewing task MEG data. The color of each waveform is based on the rank-order of the median normalized RT for each exemplar (rank is always within category). All response amplitude scales are in units of 10−14 T. The decoding onset is indicated by dashed vertical line (60 ms). The period of peak decoding is indicated by the gray shaded region extending from 120–240 ms post-stimulus onset. The bar along the x-axis indicates the stimulus duration. (TIF) [file pcbi.1004316.s003.tif]
